# Biodegradation of Microcystins during Gravity-Driven Membrane (GDM) Ultrafiltration

**DOI:** 10.1371/journal.pone.0111794

**Published:** 2014-11-04

**Authors:** Esther Kohler, Jörg Villiger, Thomas Posch, Nicolas Derlon, Tanja Shabarova, Eberhard Morgenroth, Jakob Pernthaler, Judith F. Blom

**Affiliations:** 1 Limnological Station, Institute of Plant Biology, University of Zurich, Kilchberg, Switzerland; 2 Eawag: Swiss Federal Institute of Aquatic Science and Technology, Dübendorf, Switzerland; 3 Institute of Environmental Engineering, ETH Zurich, Zurich, Switzerland; Arizona State University, United States of America

## Abstract

Gravity-driven membrane (GDM) ultrafiltration systems require little maintenance: they operate without electricity at ultra-low pressure in dead-end mode and without control of the biofilm formation. These systems are already in use for water purification in some regions of the world where adequate treatment and distribution of drinking water is not readily available. However, many water bodies worldwide exhibit harmful blooms of cyanobacteria that severely lower the water quality due to the production of toxic microcystins (MCs). We studied the performance of a GDM system during an artificial *Microcystis aeruginosa* bloom in lake water and its simulated collapse (i.e., the massive release of microcystins) over a period of 21 days. Presence of live or destroyed cyanobacterial cells in the feed water decreased the permeate flux in the *Microcystis* treatments considerably. At the same time, the microbial biofilms on the filter membranes could successfully reduce the amount of microcystins in the filtrate below the critical threshold concentration of 1 µg L^−1^ MC for human consumption in three out of four replicates after 15 days. We found pronounced differences in the composition of bacterial communities of the biofilms on the filter membranes. Bacterial genera that could be related to microcystin degradation substantially enriched in the biofilms amended with microcystin-containing cyanobacteria. In addition to bacteria previously characterized as microcystin degraders, members of other bacterial clades potentially involved in MC degradation could be identified.

## Introduction

During the last century, the anthropogenic input of nutrients into freshwaters has resulted in a distinct increase of cyanobacterial biomass in many water bodies worldwide [1]. Climate change and global warming may even increase the frequency and intensity of cyanobacterial blooms in the future [2]. Some cyanobacteria represent a major challenge for drinking water usage due to their production of microcystins (MCs), toxic secondary metabolites that affect a wide range of animals and humans [3]. The major route of human exposure to MCs is via oral ingestion, mainly due to the consumption of drinking water [4]. Even a subchronic dose of MCs in drinking water may elevate the rate of liver cancer, as was shown in China, where prevalent incidences of liver cancer correlated with MC-contaminations in drinking water [5]. Consequently, the WHO has developed a guideline for MCs in drinking water stating that an average exposure generally should be below the level of 1 µg L^−1^ [4]. *Microcystis aeruginosa* is known to form massive blooms in many lakes worldwide, and it produces MCs in high amounts. The concentrations of the intracellular MCs range between 0.3 to 15 µg L^−1^ [6] and up to 400 µg L^−1^ [7] in cyanobacterial blooms, however, occasionally high concentrations of up to 1400 µg L^−1^ were found [8]. Elimination of the MCs from drinking water is therefore highly desirable.

The largely cell-bound MCs are eliminated by removing the intact cyanobacterial cells by conventional water treatment procedures such as coagulation or flocculation [4]. However, cell damage (e.g. during the collapse of a bloom) will release toxins into water, and the above mentioned procedures will not sufficiently remove MCs from drinking water. Strategies such as powdered activated carbon [9], sediment sorption [10], or ozonisation [11] have been suggested to effectively eliminate dissolved MCs. However, these treatments are costly in terms of development and management (energy, need of chemicals) and thus not suitable for the application in developing and transient countries.

Gravity driven membrane (GDM) ultrafiltration is considered for drinking water production as a relevant alternative to common appliances [12]. GDM uses a simple set-up [13], which is inexpensive, electricity-free, easy to use, and it is already known to provide an effective barrier against pathogens, disease vectors and suspended solids [14]. Microbial activity as well as the total organic carbon content in the feed water have been shown to affect the performance of the GDM ultrafiltration without control of the biofilm formation (no backwashing or chemical cleaning) [13]. However, nothing is currently known about the possible degradation processes of intact cyanobacterial cells or of toxins such as MCs in these point-of-use membrane systems. In recent years, biodegradation by heterotrophic bacteria has been recognized as an alternative way to eliminate MCs [15]. A few bacterial isolates capable of MC degradation have been already characterized [16]. The best studied MC degrading bacteria are belonging to the *Alphaproteobacteria* such as *Sphingomonas* sp. [17], *Sphingopyxis* sp. [18], or *Novosphingobium* sp. [19]. However, only a few studies have tried to link the composition of bacterial communities in plankton [20] or in biofilms of biological drinking water treatment facilities [21] with the ability of these systems to degrade microcystins.

The objective of our study was to examine possible degradation of MCs by the microbial biofilm of a GDM ultrafiltration system. We simulated cyanobacterial blooms and their collapse (and thus the release of the cell-bound MCs into the surrounding water) and determined the ability of the microbial biofilm to remove MCs during drinking water production. We also analysed the composition of microbial assemblages of the biofilms of the GDM ultrafiltration systems by next generation sequence analyses in order to obtain information about the microorganisms that might potentially be involved in this process.

## Materials and Methods

### Cyanobacterial cultures and quantification of microcystins

Axenic cultures of Microcystis aeruginosa PCC 7806 were kept at 20°C in Cyano-medium in several Erlenmeyer flasks under constant light at 5 µmol quanta m^−2^ s^−1^ from fluorescent tubes. Fresh Microcystis aeruginosa PCC 7806 cultures were taken every three to four days from the cultivation for the ongoing experiment. The cell number of the cyanobacterial culture was determined by flow cytometry (described below), and the MC concentration was quantified by high-performance liquid-chromatography (HPLC) as followed: A volume of 5 mL of the culture was frozen at −23°C for three hours. After thawing, 7.5 mL of 100% methanol (MeOH) were added to achieve a 60% aqueous methanolic solution. The extract was centrifuged for 15 min at 25′700 g. HPLC analysis was performed on a Shimadzu 10 AVP system with photodiode array detector (PDA) and a Hydrosphere C18 column (YMC, 4.6×250 mm, Stagroma, Switzerland), using solvent A: UV-treated H_2_O containing 0.05% trifluoroacetic acid (TFA, Merck) and solvent B: acetonitrile and 0.05% TFA. A gradient was achieved by applying linear increases in two steps (solvent B from 35% to 70% in 30 min, 70% to 100% in 2 min). For the quantification procedure, calibration curves for MC-LR and [D-Asp^3^] MC-LR, the two MCs of M. aeruginosa PCC 7806 had to be established: The two MCs were isolated in high purity (>99%, HPLC) from Microcystis aeruginosa PCC 7806, and their specific molar absorption coefficient was used to prepare accurate standard solutions between 1 and 10 µg mL^−1^. The calibration curves were based on the peak area recorded at a wavelength of 239 nm. The microcystin quantification was done in duplicate and is referred to as the sum of the concentrations of MC-LR and [D-Asp^3^] MC-LR.

### Experimental setup of the Gravity-Driven-Membrane (GDM) system

**Figure 1:**
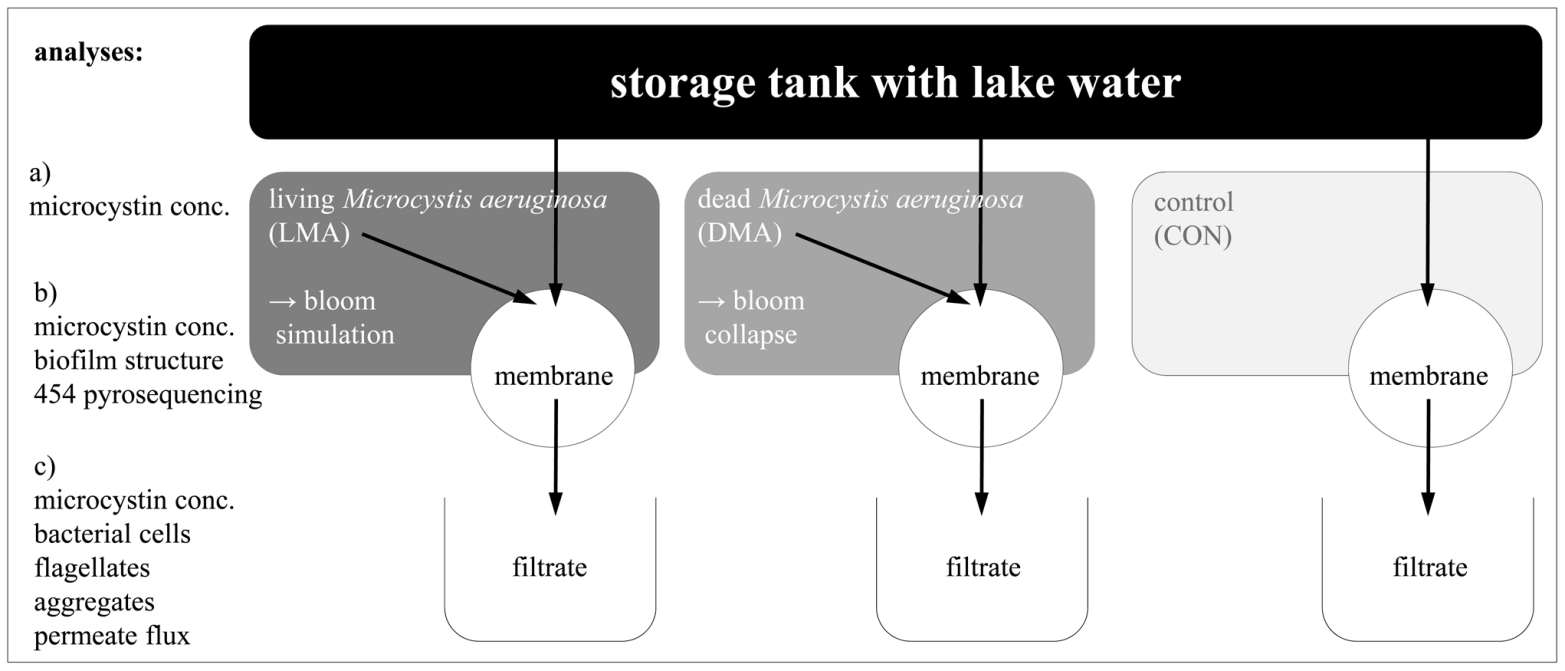
Schematic view of the gravity-driven membrane (GDM) system. Depicted are the three different operating treatments (LMA, DMA, and CON), the ultrafiltration membrane (150 kDa) and the filtrate collection. Analyses that were carried out during this study a) in the feed water, b) in/on the biofilm, and c) in the filtrate are listed on the left side of this overview.

Water from a depth of 5 m of Lake Zurich was continuously pumped by a fountain pump (Nautilus 450, Oase GmbH, Hörstel, Germany) to a storage tank (6 l volume, kept in the dark at room temperature). This storage tank was connected by silicon tubes (Saint-Gobin) to six parallel membrane modules consisting of filter holders of 48 mm inner diameter (Whatman, Maidstone, Kent, UK) and polyethersulfone ultrafiltration membranes with a 150 kDa nominal cut-off (PBHK, Biomax Millipore, Billerica, MA, USA). A hydrostatic pressure of 0.65 mbar was received by keeping the storage tank 0.65 m above the membrane surface. Overflow conditions at the storage tank guaranteed constant transmembrane pressure. Filter holders, silicon tubes and glass bottles for collection of filtrate water were autoclaved prior to experiment. Ultrafiltration membranes have been soaked in nanopure water (Bearnstead, Thermo Scientific, Basel, Switzerland) for 24 h before starting the experiment. The six membrane systems were split into three different treatments, each with two replicates (Figure 1): the control treatment (referred to as CON) received lake water only. A Microcystis bloom was simulated in two replicates: the lake water was enriched with the cyanobacterium Microcystis aeruginosa PCC 7806 (about 2×10^8^ cells) once every 24 h (treatment subsequently referred to as LMA: living Microcystis aeruginosa). A collapsing cyanobacterial bloom was simulated in the last treatment. A culture of Microcystis aeruginosa PCC 7806 was first frozen for 3 h at −20°C, and thawed before adding to the membrane system once every 24 h (referred to as DMA: dead Microcystis aeruginosa). The cyanobacterial cells were directly added above the filtration membrane module to avoid MC degradation processes in the storage tank or during tubing passages. All filter systems were kept in the dark. The filtrate water of all treatments was collected every 24 h, and the volume was determined to quantify the permeate flux (as L m^−2^ h^−1^). A subsample of 1 mL of each filtrate was fixed with 50 µl glutaraldehyde (2.5% final concentration) for cell enumeration at the flow cytometer. The rest was stored at 4°C for microcystin (MC) quantification (usually done within 24 to 48 h). The experiment was running for 21 days. At the end of the experiment, the ultrafiltration membranes were cut into three equal parts that were subjected to the following analyses: (i) One part of the filter was used to quantify MCs by HPLC that possibly remained in or attached to the biofilm on the filters. (ii) Phylogenetic analyses (454 tag pyrosequencing) were performed with the biofilm on another filter part, and (iii) the last part was used to take a closer look on biofilm structures by non-invasive methods such as Optical Coherence Tomography (OCT) (model 930 nm Spectral Domain, Thorlabs GmbH, Dachau, Germany). OCT images were analysed for average thickness and relative roughness using image analysis software developed under Matlab (MathWorks, Natick, US) [12]. To determine the exact membrane area of the pieces image analysis (Zeiss, AxioVision 4.7) was applied using a microscope (AxioImager.Z1, 1 x EC Plan-Neofluar, Zeiss) and a CCD camera (AxioCam MRm, 12 bit grayscale, 1388×1040 px, Zeiss).

### Flow cytometric enumeration of cells in the filtrate water

Samples for flow cytometry were stained with DAPI (4′,6-diamidino-2-phenylindole, 1 µg mL^−1^ final concentration) for 15 min in the dark. Subsequently, samples were analysed using an Influx V-GS cell sorter (Becton Dickinson, Inc., San Jose, CA) equipped with a UV laser (60 mW, 355 nm; CY-PS; Lightwave Electronics) for detection of DAPI fluorescence, and a blue laser (200 mW, 488 nm; Sapphire; Coherent Inc.) for scattered light and autofluorescence of flagellates. If necessary, and to avoid particle coincidence, samples were diluted with sheath fluid (2.5 g L^−1^ NaCl; filtered by 0.2 µm-pore-size). Sample volume was calculated from the analysed sample weight. Data obtained by flow cytometry were analysed with the custom-made software ViiGate 1.0a. Bacterial cells were identified using side scattered light (SSC) versus DAPI fluorescence (431 nm), flagellates were determined on the basis of SSC versus green fluorescence (531 nm). Bacterial aggregates were operationally defined by their DAPI fluorescence and scatter properties equal to or higher than that of flagellates [22]. Cyanobacterial cells of the *Microcystis aeruginosa* PCC 7806 culture were identified using SSC versus their auto-fluorescence at 692 nm (without prior staining).

### Extraction of microcystins

The entire filtrate was collected for solid phase extraction of MCs. The C18 cartridges (1 g, 60 mL, Mega Bond Elute, Varian, Agilent Technologies, Basel, Switzerland) were first equilibrated with 10% MeOH before adding the filtrate water. Afterwards, the MCs were eluted with 100% methanol. The samples were dried in a vacuum rotary evaporator at 40°C and 35 mbar. The residues were re-suspended in 1 ml 60% MeOH, the microcystin quantification was performed by HPLC in duplet. The MC concentrations in the filtrate were always measured 24 h after injection and were expressed as percentage of the MCs that were removed from the system (removal efficiency) or as MC removal rates (ug L^−1^ d^−1^).

At the end of the experiment, GDM polyethersulfone membranes were first frozen at −23°C for 3 h to isolate the MCs from the biofilm. After thawing, the biofilms were extracted twice with 5 mL 60% MeOH for 1 h, and both extracts were combined. Accordingly, the solvent was evaporated (40°C and 35 mbar), and the samples were prepared for HPLC analysis. The residues were re-suspended in 1 ml 60% MeOH, and centrifuged for 5 min at 10′000 rpm. Afterwards, the supernatants were taken for microcystin quantification by HPLC (as described above).

### 454 tag pyrosequencing analysis

Prior to the analysis, the filter parts of both replicates were pooled. The DNA extraction of the biofilm bacteria was performed using the UltraClean Water DNA isolation kit (MO BIO Laboratories, Inc.). Subsamples of 300 µl of DNA suspension (final concentration 4–10 ng µL^−1^) of all three DNA extractions (CON, LMA, and DMA) were sent to Research and Testing Laboratory, Inc. (Lubbock, TX, USA) for further processing. Partial 16S rRNA gene encoding sequences were obtained from 454 pyrosequencing (Roche FLX platform) following Assay b.9 by using the primer pair 799F and 1115R that exclusively amplify DNA of heterotrophic bacteria and exclude cyanobacteria from the process [23]. Raw data (68′981 Reads; mean raw read length 378.4 base pairs) were processed by a custom-made pipeline on a local computer cluster consisting of 16 units (each equipped with an 8 core AMD FX-8150 CPU, 16 GB RAM and a 128 GB SSD hard disk) and a separate control workstation. The program was developed in DELPHI and run under Windows 7. The processing of the raw data is extensively described elsewhere [24]. In brief, reads were denoised at the level of flowgrams according to Quince and co-workers [25]. Afterwards, quality filtering strategies were applied to finally end up with the number of 27′391 sequences (raw reads reduced by 60%) corresponding to 956 operational taxonomic units (OTUs, 3% similarity). A distance matrix was calculated and the OTUs were produced after the pairwise alignment (Needleman-Wunsch algorithm) by average linkage at similarity levels of 97%. OTUs were assigned to taxonomic entities on the level of similarity of the OTU to the most closely related sequence in the SILVA reference data base (release 109) [26]. OTUs were grouped into different levels of sequence identity: ≤3% divergence in 16S rRNA gene sequence corresponds to species level, ≤5% to genus level, and ≤10% to family level. Finally, the OTUs of the both treatments were compared with each other. Only OTUs were included that were >0.5% of sequences per sample (CON or LMA+DMA) and that occurred at least ten times more frequently in one treatment than the other. Shannon’s diversity index was estimated using the formula H′ = − Σ(P_i_ * ln P_i_), where P_i_ is the relative abundance of the sequences per sample. The index H′ is used to characterize the diversity of species or species-like units (OTUs) in a community.

## Results

### Physical parameters: Permeate Flux and structure of the biofilms

**Figure 2:**
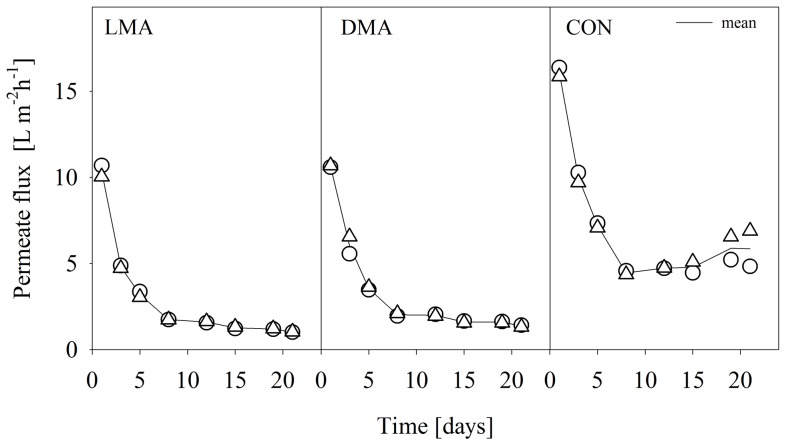
Evolution of the permeate flux. The flux is shown in L m^−2^ h^−1^ for the filtration of differently treated feed water sources (LMA, DMA, and CON according to Figure 1). The two replicates per system are shown as circles and triangles, and are connected by the mean.

Flux stabilization was observed approximately after eight to ten days of the experiment in all three treatments albeit great differences between the control and both Microcystis treatments (Figure 2). A mean flux of 4.7 L m^−2 ^h^−1^ was measured after 12 days in the CON treatment. In one of the two replicates, the flux stayed constant until the end of the experiment. In the second replicate, the flux increased slowly to 6.9 L m^−2^ h^−1^ on day 21. Accordingly, the mean thickness (as assessed by OTC measurements) of the biofilms of both control replicates at the end of the experiment were slightly different. The biofilm of the first replicate had a thickness of about 125 (±23) µm; the biofilm of the second replicate was 96 (±17) µm thick. The second replicate was less heterogeneous than the first replicate, but both exhibited low relative roughness values of 0.49 and 0.35, respectively. The permeate flux in both Microcystis treatments showed a similar trend. Stabilization could be observed at a mean flux of 1.6 L m^−2 ^h^−1^ in the LMA and of 2.0 L m^−2 ^h^−1^ in the DMA treatment. In both treatments, mean permeate flux decreased further to 1.0 L m^−2^ h^−1^ and 1.36 L m^−2^ h^−1^, as measured at the end of the experiment. Thus, the mean flux in the Microcystis replicates was about 80% lower than in the CON treatment. Biofilms in the DMA treatment were about six to seven times thicker as in the CON treatment with values of 625 (±33) µm and 796 (±29) µm for both replicates. Unfortunately, a quantification of the biofilm thickness in the LMA treatment could not be carried out.

### Microcystin removal

**Figure 3:**
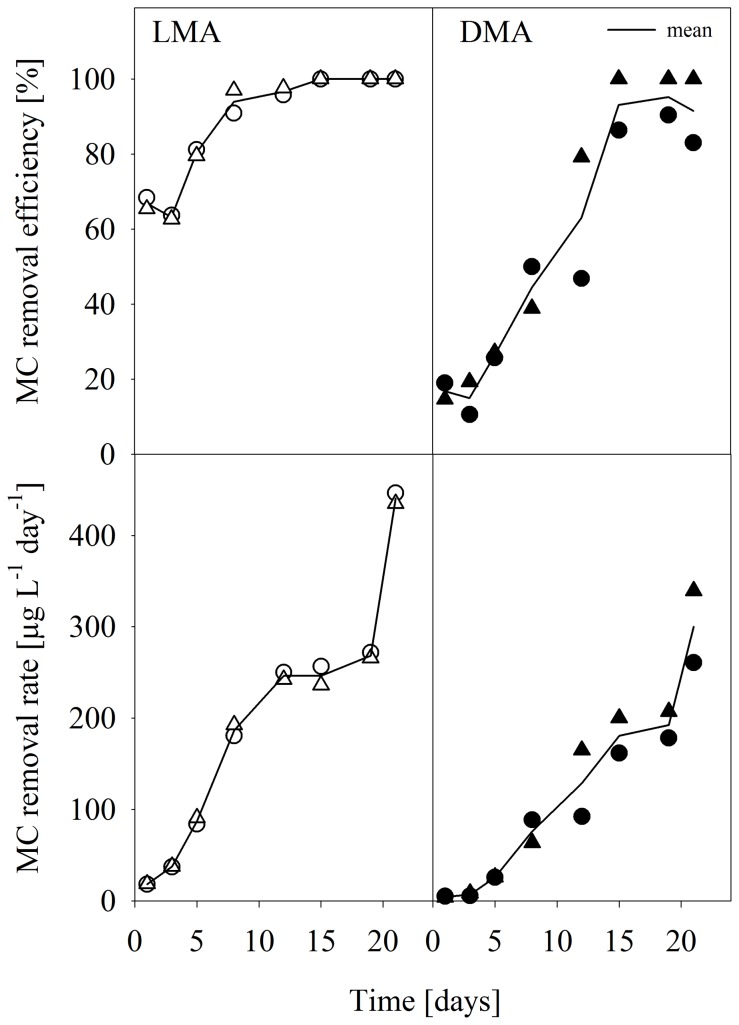
Microcystin removal and removal rates. Time course of the microcystins (MCs) removal efficiency of the GDM system (upper panel) and the MC removal rate (lower panel) in the two *Microcystis* treatments (LMA and DMA according to Figure 1). The two replicates per system are shown as circles and triangles, and are connected by the mean.

The microcystin removal efficiency of the biofilms was calculated from the amount of MCs that was injected via living or dead Microcystis cells into the systems and the amount that was measured 24 hours later in the filtrates (Figure 3, upper panel). In the LMA treatment, both replicates were working similarly, showing already high removal efficiency of almost 70% at the beginning. After 10 days of the experiment, the biofilms of both replicates showed nearly 100% removal efficiency; complete removal was achieved after 15 days and remained constant until the end of the experiment. Both replicates of the DMA treatment started with a low removal efficiency of about 10% during the first three days of the experiment. One of the replicates developed a biofilm that was able to remove the MCs completely after 15 days. The MC removal efficiency of the biofilm of the second replicate decreased again to less than 80% (Figure 3, upper panel). The MC removal rates in both treatments increased during the course of the experiment up to 440, respectively 300 µg L^−1^ d^−1^ (Figure 3, lower panel).

Altogether, 4.6×10^9^ living or dead cells of M. aeruginosa (containing 262.5 µg MCs) were added to each MC-replicate of the GDM systems throughout the duration of the experiment (Table 1). About 10% (27 µg) of the added MCs were found again in the filtrates of each replicate of the LMA treatments during the entire experiment, 96.5 µg (36.8%) were found on the LMA filters at the end of the experiment. Thus, 140 µg of MCs were removed in both replicates during the course of the experiment. Only 0.5 µg MC was found on the filter in the DMA treatments, 99 µg, respectively 121 µg have been collected in the DMA filtrates of both replicates; 141–163 µg of the MCs were degraded during the course of the experiment.

**Table 1:** Microcystin concentrations.

	LMA		DMA	
	Replicate 1	Replicate 2	Replicate 1	Replicate 2
Total amount of MCs injected over 21 days	262.5 (100)	262.5 (100)	262.5 (100)	262.5 (100)
Amount of MCs in the filtrate on day 21	0 (0)	0 (0)	0 (0)	2.9 (1.11)
MCs on the filter at the end of the experiment (on day 21)	96.5 (36.8)	96.5 (36.8)	0.5 (0.2)	0.5 (0.2)
Total amount of MCs in the filtrates, collected for 21 days	28 (10.6)	26 (9.9)	99 (37.7)	121 (46.1)
MCs loss	138 (52.6)	140 (53.3)	163 (62.1)	141 (53.7)
Threshold concentration of less than 1 µg L^−1^ was reached on day	15	15	15	Not reached

Overview about the amount of MCs [µg; (%)] totally injected, found on the biofilm or in the filtrate, and the estimated loss of MCs during the experiment. Last row shows the day, at which the threshold concentration of less than 1 µg L^−1^ was reached (LMA and DMA according to Figure 1).

### Cell numbers

**Figure 4:**
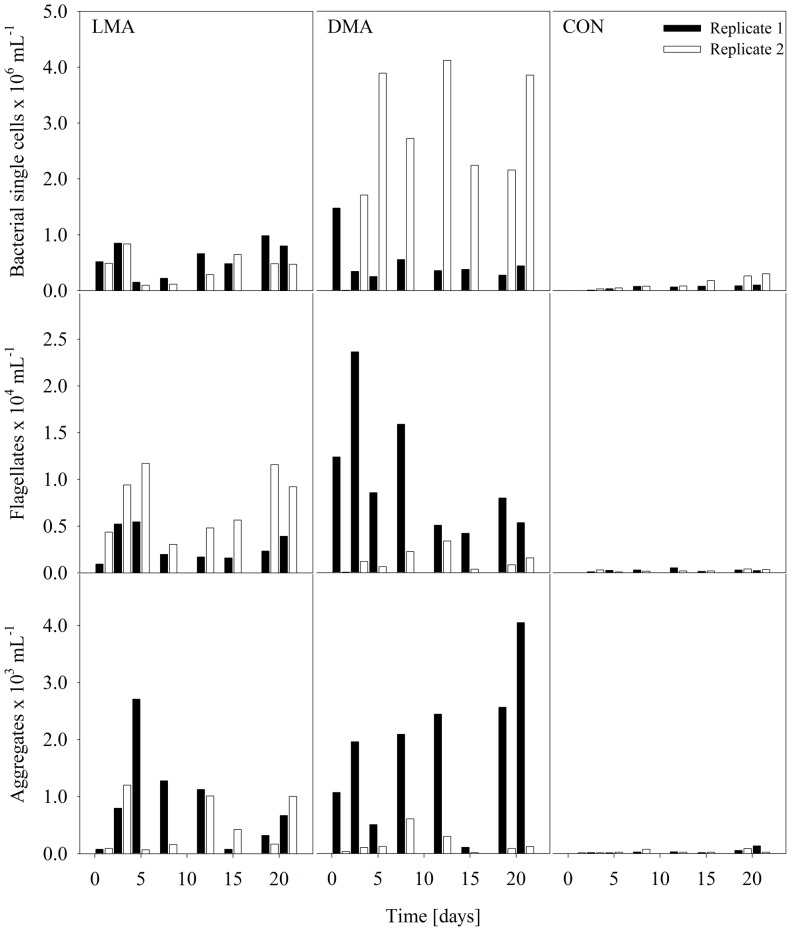
Cell abundances in the filtrate. Regrowth of bacterial single cells (upper panel), flagellates (middle panel) and flagellate-inedible bacterial aggregates (lower panel) in the filtrate of the three different treatments (LMA, DMA, and CON according to Figure 1).

Bacterial single cell numbers in the CON filtrates constantly increased during the course of the experiment (Figure 4, upper panel) up to 0.1 and 0.3×10^6^ cells mL^−1^, respectively. Similarly, flagellate and bacterial aggregate numbers increased slowly, but stayed on comparable low mean levels of 280 flagellates mL^−1^ and 78 aggregates mL^−1^ on day 21 of the experiment (Figure 4). In contrast, both MC treatments showed higher cell numbers as compared to the CON treatment. Between 0.8 and 0.5×10^6^ bacterial single cells mL^−1^ were found in both replicates of the LMA treatment at the end of the experiment. Comparable numbers of bacterial single cell numbers were determined also in the replicate 1 of the DMA treatment (0.4×10^6^ mL^−1^). The bacterial single cell numbers in the corresponding second replicate of the DMA treatment were about ten times higher (3.9×10^6^ mL^−1^). Flagellate numbers in the filtrates of the LMA treatment were comparable high at the end of the experiment (mean value 0.67×10^4^ mL^−1^), as well as bacterial aggregates (mean value 0.8×10^3^ mL^−1^). Comparable numbers of flagellates were found in the filtrate of the first replicate of the DMA treatment (0.54×10^4^ mL^−1^) as well as the highest amount of aggregates (4.0×10^3^ mL^−1^) at the end of the experiment. However, rather low numbers of flagellates and aggregates were found in the second replicate of the DMA treatment, 0.15×10^4^ mL^−1^ and 0.1×10^3^ mL^−1^, respectively (Figure 4).

### Phylogenetic analyses

**Figure 5:**
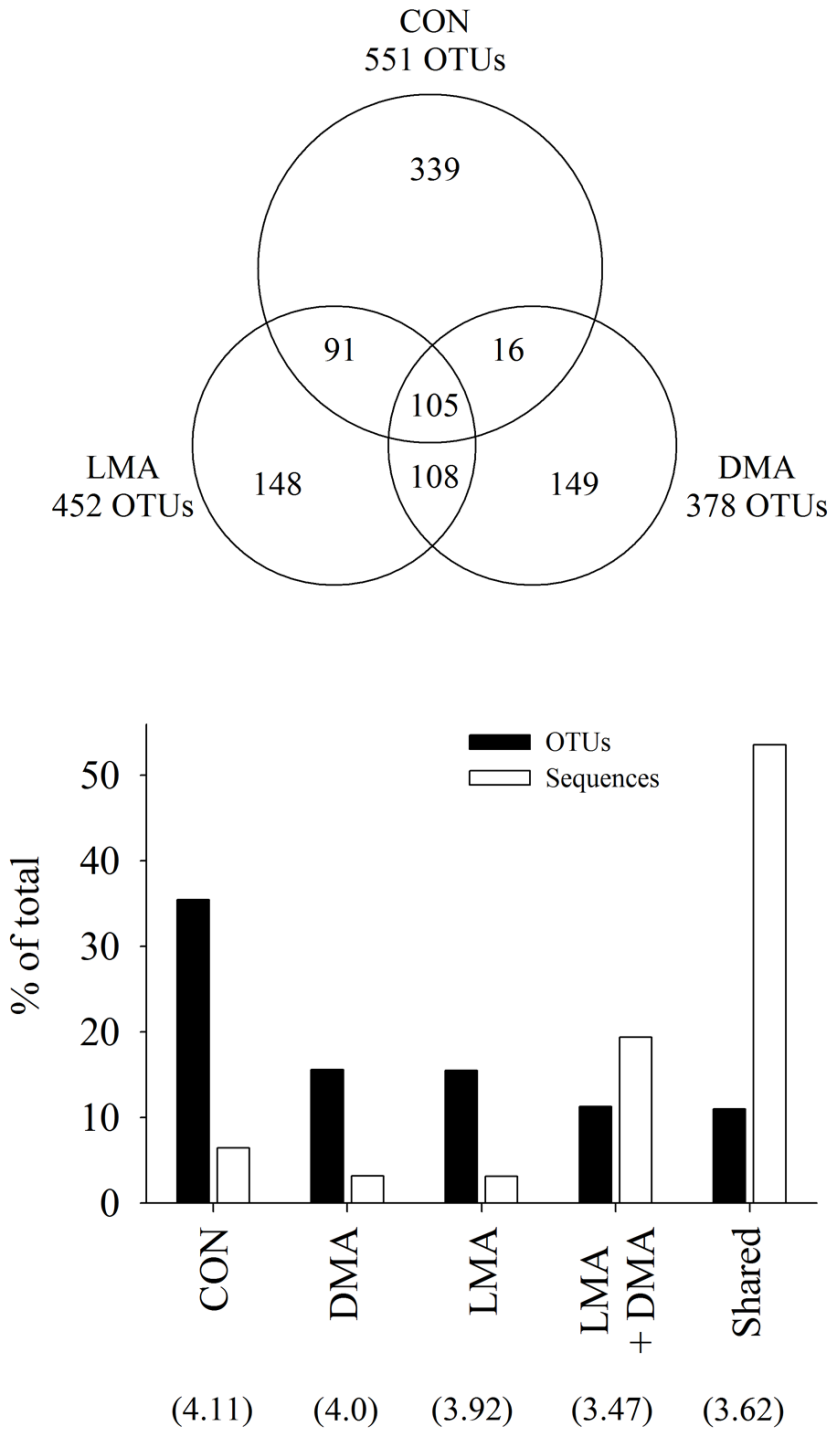
Overview of microbial diversity analysis. Upper panel: Venn diagram of shared OTUs among the biofilms of the three treatments. Lower Panel: percentage of operational taxonomic units (OTUs) and sequences found in the different treatments. Number in brackets marks the Shannon’s diversity index (*H*′) for each treatment (LMA, DMA, and CON according to Figure 1).

A total of 27′391 sequences, assigned to 956 OTUs, were evaluated after removing low quality reads and chimeric sequences. For the bacterial communities of the MC-treated biofilms, about 452 OTUs (8′064 sequences; LMA) and 378 OTUs (8′048 sequences; DMA) were determined (Figure 5A); slightly more sequences were obtained for the CON bacterial community (11′279 sequences; 551 OTUs). The bacterial communities of the three biofilms shared only 11% of all OTUs (105 OTUs), but comprised 54% of all sequences (Figure 5A and 5B). The OTUs therein were large, and consisted of 140 sequences on average. This core community consisted predominantly of Sphingobacteriales (Bacteroidetes; 48% of the shared OTUs) and Comamonadaceae (Betaproteobacteria, 45% of the shared OTUs). The CON treatment consisted of the largest amount of OTUs (35% of all OTUs) that were unique to this special treatment, but comprised only 1764 sequences (5.2 sequences per OTU). LMA and DMA shared 108 OTUs, the average sizes of these OTUs were about 50 sequences per OTU (Figure 5A and 5B).

Most of the sequences in the combined LMA and DMA (MA) communities received taxonomic assignments at least to the genus level (93% in the LMA treatment, 96% in the DMA treatment), affiliation to families could be determined for 97%, respectively 99% of these sequences. However, only 44% of all sequences in the CON communities were assigned to the genus level, and 52% to the family level. With a high divergence in similarity of more than 12% to the closest known relative, two large OTUs were assigned to either Myxococcales (1′990 sequences) or to Fibrobacteres (2′357 sequences).

### Bacterial taxa favoured by Microcystis addition

**Figure 6:**
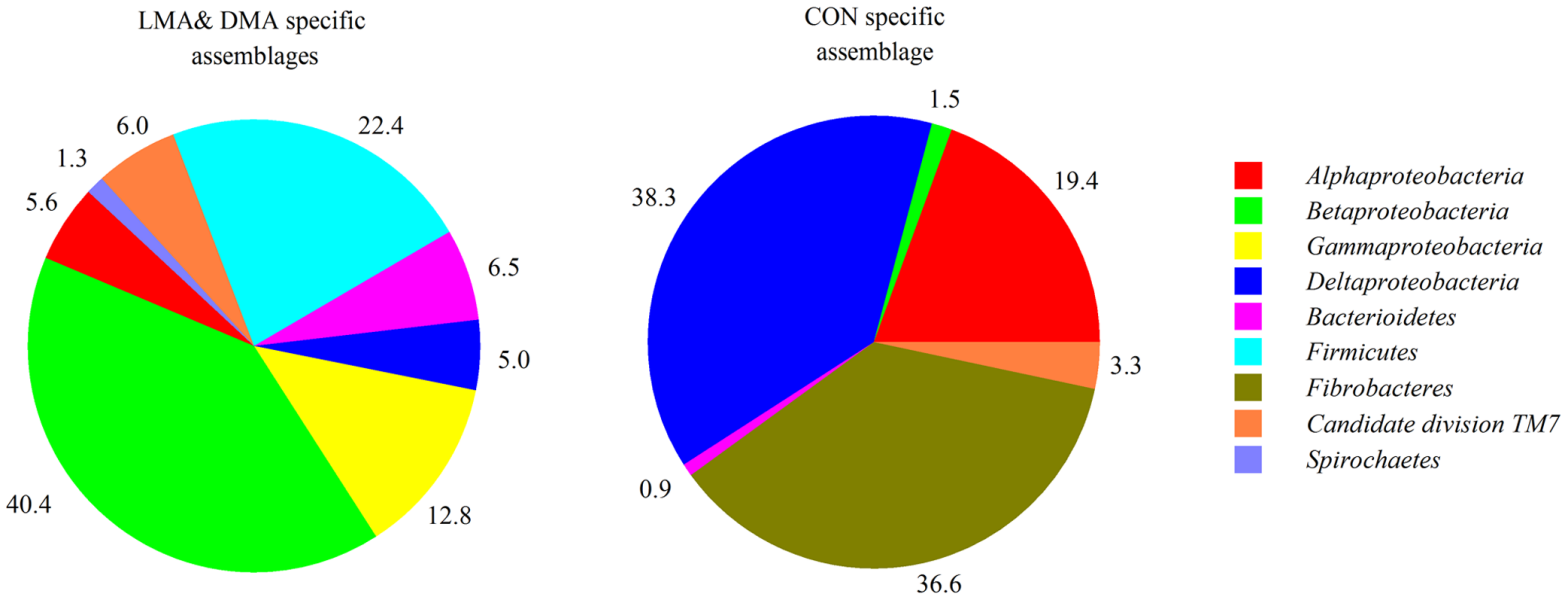
Phylogenetic composition of bacterial biofilms. Phylogenetic composition [%] of bacteria specific for the (combined) LMA and DMA assemblage (left) and for the CON (right) assemblage. Specificity for either assemblage was defined by the size of the OTU (>0.5% of sequences per sample) and by a ten times higher occurrence in one category than the other (LMA, DMA, and CON according to Figure 1).

Addition of Microcystis cells led to compositional differentiation between the communities of both MC-treated and the CON biofilms. Over one third (38.3%) of all sequences in the CON assemblage was affiliated with Deltaproteobacteria, a class that was significantly less abundant in the combined LMA and DMA (MA) communities (5.0%) (Figure 6). The Fibrobacteres (36.6%) and the Alphaproteobacteria (19.4%) were the second and third most abundant taxa affiliated with the CON assemblage, both taxa were underrepresented in MA assemblage as well. These three classes comprised almost 95% of all sequences in the CON assemblage. However, more than one third (40.4%) of all sequences in the MA assemblage was affiliated with Betaproteobacteria that were found only marginally in the CON communities. Firmicutes (22.4%) and Gammaproteobacteria (12.8%) were the second and third most abundant taxa in the MA assemblage, but were not found in the communities of the CON treatment. These three classes comprised 75.6% of all sequences. Additionally, minor fractions of Candidate division TM7 and Bacteroidetes were present in both assemblages, Spirochaetes only to a minor extent in the MA assemblage (1.3%).

Most of the bacterial genera found in the CON assemblage were typically isolated from microbial biofilms in drinking or freshwater reservoirs or pipelines, such as Hirschia, Phenylobacterium, or Comamonas. Haliangium or the Myxococcales were present in anaerobic filter sediments or suboxic freshwater ponds (Table S1 in File S1). The Fibrobacteres were isolated before from upper sediment layers and biofilm samples. Bacterial sequences affiliated with Sphingomonas containing MC degradation proteins were only found in the CON assemblage, where they made up 12% of all sequences (Table S1 in File S1). Other genera of the Alphaproteobacteria that contain MC degradation proteins or MC dependent proteins have been found only in the MA assemblage, such as Azospirillum or Magnetospirillum, as well as the Rheinheimera (belonging to the Gammaproteobacteria) or Spirochaetales (Table S2 in File S1). Some other bacterial genera have been repeatedly found in the Planktothrix layer of Lake Zurich, such as Variovorax (5.4% of all sequences in the MA assemblage) or unknown genera belonging to the Sphingobacteriales (6.5%). Paucibacter known to degrade MCs has been only found in the MA assemblage (3.2%). The Firmicutes (22.4% of all sequences of the MA assemblage) comprised mainly of Acidaminobacter that are typical inhabitants of suboxic freshwater ponds.

## Discussion

### Performance of the GDM system and fate of microcystins

Two factors have a strong impact on the performance of GDM systems: the composition of the microbial community (bacteria and predators) and the organic carbon content of the feed water [12]. In the latter study, the almost complete absence of metazoan organisms resulted in smooth and homogeneous biofilm structures. In our study, this was indicated by the comparably low values of relative roughness of both CON replicates. In the absence of predation, the total organic carbon (TOC) content governs the permeability of the biofilms. The decrease of the permeate flux with increasing TOC content is due to, both, a higher accumulation of particulate matter (originated from the influent) and a higher bacterial growth because of the higher nutrient loads upon cell lysis [12]. Cyanobacterial blooms are such a source of high TOC. The enormous load of cyanobacterial biomass in both Microcystis treatments resulted in the massive increase in biofilm thickness. Thus, both Microcystis treatments lost 80% of their performance, which was shown by a much lower flux and permeate, respectively.

At the same time, these biofilms were able to degrade MCs. A complete removal of the MCs by the biofilms in our study could be achieved after 15 days, which was one week after the stabilization of the flux, i.e., after the development of a stable biofilm. It is conceivable that after that time bacteria capable of the degradation of MCs had accumulated on the biofilm. The key role of the bacterial biofilm in MC degradation can be confirmed by comparing the performance of the GDM system during the initial phase of the experiment and after flux stabilization. A reduction of 10% during the first three days of the experiment (Figure 3) cannot be attributed to the not yet developed biofilm but rather to adsorption processes, either to the filtration units or to cyanobacterial cells that retained on the filter membrane.

However, the potential of biofilms to remove MCs does not only depend on the presence and fast accumulation of possible MC degrading bacteria, but also on their efficiencies to biodegrade these MCs. Degradation rates of various bacterial strains might range between 1.5 up to several 10′000 µg L^−1^ d^−1^ [16] Already after 15 days, the added MCs were completely removed. In our study, MC removal rates increased constantly after a short lag phase, but did not reach a plateau. Therefore, the degradation capability of the aged biofilm might have further increased and even higher concentrations of MCs might be successfully degraded. The previous natural exposure of the feed water to cyanobacteria and MCs (as is the case for water from Lake Zurich) seems to be a significant driver for a fast performance of a MC degrading biofilm and thus its accelerated maturation [27]. Lake Zurich contains large populations of Planktothrix rubescens, a filamentous microcystin-producing cyanobacterium, which is frequently found in natural pre-alpine lakes [2]. It should be noted that the biofilm in our study was selectively grown under Microcystis bloom conditions. It remains to be investigated, how a bacterial biofilm without prior presence of MCs would respond to Microcystis addition. Another interesting aspect, which was beyond the scope of our experiment, would be the comparison of the bacterial composition in the biofilms by comparing MC-producing with MC-non-producing strains. This would allow for a more specific assessment, which shifts in microbial community compositions were caused by other substrates provided by the addition of cyanobacterial biomass.

### Drinking water quality

Pro- and eukaryotic microbes can regrow during drinking water treatment and the distribution of non-chlorinated potable water [28]. The total bacterial cell counts in tap and mineral water may reach values between 1.5×10^5^ to 5×10^5^ cells mL^−1^ [29], [30]. In our study, bacterial abundances in the CON treatment were within this range. Bacteria most likely regrew in biofilms underneath the filter membranes or on materials downstream the filtration process and dropped into the filtrate [29]. Inadequate disinfection, hydraulic retention time, flow regime, pipe material, temperature, source of water or corrosion could lead to the development of a microbial biofilm. However, excessive regrowth in drinking water supply systems can be also triggered by nutrient introduction. It is conceivable that the massive load of cyanobacterial biomass in our study led to higher bacterial cell numbers, not only under the membrane filter but also in the filtrate. The increased availability of various substrates released from broken cells in the DMA treatment resulted in even higher cell numbers in the corresponding filtrates. As the drinking water quality may be deteriorated by the presence of pathogenic bacteria, an adequate storage of the drinking water is important to avoid re-contamination [31]. The focus in our study was on the removal of cyanobacterial toxins. However, the effect of other components of cyanobacterial cells that enhance regrowth remains to be investigated.

Drinking water storage or distribution systems represent functional ecosystems with well-established and structured microbial communities [32]. The increasing numbers of bacteria support the succession of protists: on average 10^5^ cell L^−1^ were found in the water phase and 10^3^ cells cm^−2^ in different biofilms [32]. As a result, some bacteria may aggregate to overcome the predation pressure by protists as it was shown in field and laboratory studies before [15]. The regrowth of bacteria underneath the filter membranes or on materials downstream the filtration process of the LMA treatments in our study stimulated the growth of bacterivorous flagellates, followed by an increase in bacterial aggregates (Figure 4). Interestingly, the DMA replicates showed two different responses: high numbers of free-living planktonic bacteria were established in the absence of flagellates in one replicate of the DMA treatment, whereas the numbers of free-living bacteria in the second replicate were low, as bacteria either were grazed by flagellates or formed aggregates in order to resist predation. This illustrates that there might be variability in the primary microbial assemblages of such filtrates that may have consequences for the development of drinking water quality.

### Differences in microbial biofilms

Comparative phylogenetic analyses of 16S rDNA has increased our understanding of microbial diversity in environmental samples, since only few of the identifiable major phyla within the domain Bacteria have cultivable representatives [33]. Many of these uncultivated bacteria are found in diverse habitats in extraordinarily high abundances and might be at best only remotely related to strains that have been characterized by phenotype or by genome sequencing [33]. This in turn implies that there is very limited understanding of their respective physiologies, e.g., their substrate degradation potential. Calculating distances to sequences in a well-curated database [26] allowed us to classify OTUs to the closest taxonomic level. The CON treatment was the most diverse of all treatments, as reflected by the high number of small OTUs and an H′ index of 4.11. With the exception of river and stream habitats [34] biofilms that occur under more oligotrophic conditions (as in the CON treatment) seem to be rather understudied. As a consequence, more “exotic” species were found that had a high distance to the closest known relatives (CKR), such as uncultured Myxococcales (11.3–12.1% distance to CKR) and Fibrobacteres (12.7% distance to CKR) (Table S1 in File S1).

The massive load of cyanobacterial biomass selected for a few large OTUs (H′ = 3.47 of the combined MA) and the bacterial taxa represented by these OTUs were from a more “known” bacterial diversity (Table S2 in File S1). An intriguing example for typical freshwater bacteria that were present in our MA samples is a set of OTUs with <3% distance to known culturable genera, such as Azospirillum, Pelomonas, or Undibacterium (Table S2 in File S1). Moreover, oxygen subsaturation in the thick biofilms of the MA treatment was suggested by the presence of obligate anaerobic bacteria such as Desulfovibrio, Acidaminobacter, Fusibacter, or Spirochaetales. The ability to remove MCs appears to be not only common for aerobic bacteria, but can also be a feature of anaerobic microorganisms (not otherwise specified) that were found in lake sediments or sediments of water recharge facilities [35]. It seems that several Spirochaeta contain the MC-LR degradation protein MlrC (Table S2 in File S1). However, further studies are needed to determine possible MC degrading bacteria in anoxic environments. Interestingly, Bacteroidetes such as Sphingobacteriales that are common in the Planktothrix layer of Lake Zurich [36] were also present in high proportions in the MA treatments only. The natural co-occurrence of these bacteria with MC-producing cyanobacteria might indicate a possible and so far unknown role in the MC-degradation process.

Previous investigations have suggested the Sphingomonadales (Alphaproteobacteria) being the major MC degraders in aquatic environments: genetic studies on Sphingomonadaceae revealed the distinct gene cluster mlrABCD to be involved in MC removal [37], because it encodes for an enzymatic ring cleavage and thus a linearization of the MCs. However, during a massive cyanobacterial bloom in Lake Erie only ∼1% of the total bacterial community could be attributed to Sphingomonadales [38] and also the metagenomic identification of bacterioplankton taxa involved in MC degradation revealed only a minor importance of these bacteria [20]. Our data also suggest that Sphingomonadales may not necessarily be relevant in MC degradation, as they only enriched in the CON treatment (Figure 6, Table S1 in File S1).

Our data indicate that Betaproteobacteria may be more important amongst the major MC degradation bacteria as they constituted the major fraction (>40%) in the MA biofilms but were hardly found in the CON assemblages. OTUs were found in high quantities that were closely related to Paucibacter, capable of degrading MCs [39] and Variovorax, containing MC-LR degradation proteins, (Table S2 in File S1). The importance of Betaproteobacteria (mainly Burkholderiales and Methylophilales) has already been suggested before based on laboratory microcosms experiments amended with MCs [38]. Interestingly, recent studies revealed that Betaproteobacteria such as Methylophilales were capable of degrading MCs but lacked the mlr cluster, thereby providing an alternative and so far unknown means of MC removal [20]. Studies focussing on the detection of the mlrA gene as the only marker for MC degradation bacteria might therefore underestimated the possible presence of other MC degrading bacterial taxa [16], [20], [21].

## Conclusion

- We demonstrated that GDM ultrafiltration systems provide a fast and efficient way to remove MCs from drinking water. However, it should be noted that complete MC degradation only took place one week after establishment of a stable biofilm.
- Addition of live or dead Microcystis cells led to remarkable differences between the bacterial communities of both MC-treated and the CON biofilms.
- Betaproteobacteria were identified as potentially important taxa for MC degradation in the MA biofilms. Additionally, Spirochaeta and Bacteroidetes such as Sphingobacteriales were enriched in these biofilms, and might indicate their so far unknown role in the MC degradation process.

## Supporting Information

File S1 File S1 contains two supplemental tables: **Table S1,** **Phylogenetic composition.** Affiliation of bacteria in the CON assemblage (OTUs specific for CON treatment), number of OTUs and sequences, and phylogenetic distances of OTUs and associated sequences in the CON assemblage to the most closely related genotype in the SILVA reference database. **Table S2,** **Phylogenetic composition.** Affiliation of bacteria in the LMA & DMA assemblage (OTUs specific for the *Microcystis* treatment), number of OTUs and sequences, and phylogenetic distances of OTUs and associated sequences in the LMA & DMA assemblage to the most closely related genotype in the SILVA reference database.
(DOCX)
