# Supplementary material for: Biodegradation of Microcystins during Gravity-Driven Membrane (GDM) Ultrafiltration
Source: PLoS One. 2014 Nov 4;9(11):e111794. doi: 10.1371/journal.pone.0111794 (PMC4219780; doi:10.1371/journal.pone.0111794)
Supplement: File S1 — contains two supplemental tables: Table S1, Phylogenetic composition. Affiliation of bacteria in the CON assemblage (OTUs specific for CON treatment), number of OTUs and sequences, and phylogenetic distances of OTUs and associated sequences in the CON assemblage to the most closely related genotype in the SILVA reference database. Table S2, Phylogenetic composition. Affiliation of bacteria in the LMA & DMA assemblage (OTUs specific for the Microcystis treatment), number of OTUs and sequences, and phylogenetic distances of OTUs and associated sequences in the LMA & DMA assemblage to the most closely related genotype in the SILVA reference database. (DOCX) [file pone.0111794.s001.docx]

Biodegradation of Microcystins during Gravity-Driven Membrane (GDM) Ultrafiltration

Esther Kohler^1^, Jörg Villiger^1^, Thomas Posch^1^, Nicolas Derlon^2^, Tanja Shabarova^1^, Eberhard Morgenroth^2,3^, Jakob Pernthaler^1^, Judith F. Blom^1*^

^1^ Limnological Station, Institute of Plant Biology, University of Zürich, Seestrasse 187, CH-8802 Kilchberg, Switzerland

^2^ Eawag: Swiss Federal Institute of Aquatic Science and Technology, Überlandstrasse 133, CH-8600 Dübendorf, Switzerland

^3^ Institute of Environmental Engineering, ETH Zürich, CH-8093 Zürich, Switzerland

*Corresponding author, phone: +41 44 634 9212, fax +41 44634 9225, E-mail: blom@limnol.uzh.ch

**File S1**

**Table S1.** **Phylogenetic composition.** Affiliation of bacteria in the CON assemblage (OTUs specific for CON treatment), number of OTUs and sequences, and phylogenetic distances of OTUs and associated sequences in the CON assemblage to the most closely related genotype in the SILVA reference database.

| **Affiliation** | **nOTUs** |  | **No. of sequences**  **in control**  **(LMA+DMA)** | **Distance to closest known relative** | **Accession number of close relative** | **Comments** |
| --- | --- | --- | --- | --- | --- | --- |
| **Alpha-**  **proteobacteria** | 1 | **Caulobacterales** *Hirschia* | 198 (14) | 4.7 % | EF018616 | Isolated from microbial biofilms, drinking water reservoirs, freshwater reservoirs, membrane bioreactors |
|  | 1 | *Phenylobacterium* | 132 (0) | < 3 % | HM186916 | Isolated from microbial biofilms, drinking water biofilms, freshwater reservoirs |
|  | 1 | **Rhizobiales**  Uncultured bacterium | 66 (3) | 4.8 % | EU133543 | Isolated from lake water, ground water, drinking water treatment plant, epilithic biofilms, water filters |
|  | 1 | Uncultured bacterium | 79 (0) | 4.6 % | EF018119 | Isolated from soil, forest soil, rice fields |
|  | 1 | **Sphingomonadales**  *Sphingomonas* | 237 (1) | < 1 % | HM921106* | Isolated from a drinking water treatment plant; contains MC degradation proteins (MlrGenes) / Microcystinase^1^ |
|  | 1 | Uncultured bacterium | 534 (43) | < 3 % | DQ664243 | Isolated from freshwater ponds, biofilms in drinking water pipelines, often found in Lake Zürich^2^ |
| **Beta-**  **proteobacteria** | 1 | **Burkholderiales**  Comamonas | 94 (1) | 6.4 % | GU208362 | Isolated from lake sediment, microbial mats, drinking water pipelines, lake water |
| **Delta-**  **proteobacteria** | 1 | **Myxococcales**  *Haliangium* | 131 (0) | 4.2 % | FJ660578 | Isolated from anaerobic fermentation filter sediments; grassland, soil, prairie |
|  | 2 | Uncultured *Myxococcales* | 2’059 (1) | 12.1 % | AB530188 (2x) | Isolated from aquatic ecosystems, sediment, suboxic freshwater ponds |
|  | 1 | Uncultured *Myxococcales* | 273 (0) | 11.3 % | EU134500 | Estuary sediments, microbial mats, soil |
| **Bacteroidetes** | 1 | **Cytophagales**  Uncultured bacterium | 58 (3) | < 1 % | FM201078 | Degrade biomacromolecules like proteins, chitins, pectin, cellulose; are ubiquitous, abundant, play a major role in the turnover of matter in the nature |
| **Fibrobacteres** | 1 | **Fibrobacteria**  Uncultured bacterium | 2‘357 (29) | 12.7 % | FJ516973 | Isolated from marine sediments, upper sediment layers, biomat samples |
| **Candidate division TM7** | 1 | Uncultured bacterium | 214 (0) | < 3 % | HM362529 | Isolated from karst spring water, lake, river, municipal waste water sludge |
| **Sum**  **% total** | **14** |  | **6‘432** |  |  |  |
|  | 1.5 |  | 23.5 |  |  |  |

^1^ Protein Knowledgebase (UniProtKB); ^2^ Michaela M. Salcher (personal communication); * bacterial genus provided genomic reference for MC degradation

**Table S2.** **Phylogenetic composition.** Affiliation of bacteria in the LMA & DMA assemblage (OTUs specific for the *Microcystis* treatment), number of OTUs and sequences, and phylogenetic distances of OTUs and associated sequences in the LMA & DMA assemblage to the most closely related genotype in the SILVA reference database.

| **Affiliation** | **nOTUs** |  | **No. of sequences**  **LMA+DMA**  **(CON)** | **Distance to closest known relative** | **Accession number of close relative** | **Comments** |
| --- | --- | --- | --- | --- | --- | --- |
| **Alpha-**  **proteobacteria** | 1 | **Rhodospirillales**  *Azospirillum* | 90 (0) | < 3 % | AB545627* | Isolated from soil, rizophere; fix nitrogen; contains MC-LR degradation protein MlrC/microcystin dependent protein^1^ |
|  | 1 | Uncultured *Azospirillum*^+^ | 132 (0) | < 3 % | GU305783* | Isolated from lake water, waste water soil, PAH contaminated soil, perchlorate contaminated sites; contains MC-LR degradation protein MlrC/microcystin dependent protein^1^ |
|  | 1 | Uncultured *Magnetospirillum* | 87 (0) | 4.1 % | JF490037* | Isolated from freshwater environment, freshwater microcosms, drinking water supply, anaerobic degradation of aromatic compounds, perchlorate reducing bacteria; contains Microcystin-dependent protein^1^ |
|  | 1 | **Rickettsiales**  Uncultured *Can. Odyssella* | 80 (8) | < 3 % | FJ437938 | Isolated from freshwater, high mountain lake biofilm, karst spring water, industrial biofilms |
| **Beta-**  **proteobacteria** | 1 | **Burkholderiales**  *Paucibacter* | 226 (11) | 5.1 % | FM872743* | Isolated from lake sediment ; capable of degrading MCs and nodularin^2^ |
|  | 2 | *Pelomonas* | 1’326 (21) | 0.95; 4.6 % | FM886906 JN869001 | Isolated from zooplankton, lake water aggregates, freshwater habitats, sediment, water; capable of degrading aromatic compounds |
|  | 1 | *Undibacterium* | 695 (0) | < 3 % | AF236013 | Isolated from drinking water biofilms; opportunistic; found in red water events in water distribution systems^3^ |
|  | 1 | *Variovorax* | 378 (14) | 3.8 % | HM773531* | Isolated from Lake Zürich *Planktothrix* layer, drinking water, drinking water biofilms; contains MC-LR degradation protein MlrC ^1^ |
|  | 1 | **Rhodocyclales**  *Dechloromonas* | 200 (0) | < 3 % | AB237671 | Isolated from anaerobic/aerobic interface of drinking water biofilms; facultative anaerob, reduce (per)chlorate |
| **Gamma-**  **proteobacteria** | 2 | **Chromatiales**  *Rheinheimera* | 578 (2) | 3.8; 4.1 % | AFHI01000053* GU293185 | Isolated from marine, brackish, freshwater environments; related genus can degrade aromatic compounds; contain Microcystin-dependent protein^1^ |
|  | 1 | **Xanthomonadales**  *Dokdonella* | 233 (6) | < 1 % | AM981200 | Isolated from drinking water networks, marine, brackish, freshwater environments; related genus can degrade aromatic compounds |
|  | 1 | *Silanimonas^+^* | 80 (0) | < 1 % | AY557615 | Isolated from lake water, ground water, spring water, biomats |
| **Delta-**  **proteobacteria** | 1 | **Bdellovibrionales**  *Bdellovibrio* | 146 (0) | < 3 % | AY168736 | Isolated from aresenite oxidizing biofilms, soil, water, sludge, sediment; predatory bacteria |
|  | 1 | *Bacteriovorax* | 93 (0) | 4.9 % | AJ288899 | Isolated from microbial mats, lake water, soil, freshwater pond bacterioplankton, predatory bacteria |
|  | 1 | **Desulfovibrionales**  *Desulfovibrio* | 110 (0) | < 1 % | AY928231 | Isolated from soil, water, sludge, sediment; sulfate reducer, aerotolerant |
| **Bacteroidetes** | 2 | **Sphingobacteriales**  Uncultured *Sphingobacteriales* (env. OPS 17) | 457 (6) | 1.3; 4.2 % | FJ849086 AF513095 | Isolated from freshwater lakes, cyanobacterial mats; common in Lake Zürich *Planktothrix* layer (12.5m depth)^4^ |
| **Spirochaetes** | 1 | **Spirochaetales**  uncultured *Spirochaetales*^‡^ | 92 (0) | 3.5 % | GU454906* | Isolated from anaerobic bioreactors, activated sludge; several *Spirochaetales* contain MC-LR degradation protein MlrC^1^ |
| **Firmicutes** | 4 | **Clostridiales**  *Acidaminobacter* | 1‘369 (0) | < 3 % | AB247468 (2x) AF255644 HQ003641 | Isolated from a suboxic freshwater pond, polychlorinated aquifers; fermenting bacterium; chlorinated compound – dechlorination consortium |
|  | 1 | *Fusibacter* | 198 (0) | 5.6 % | AJ307951 | Isolated from microbial mats, marine; fermenting bacterium |
| **Candidate division TM7** | 1 | uncultured phylum | 416 (0) | < 3 % | AB476245 | Isolated from microbial mats, microbial biofilms |
| **Sum**  **% total** | **26** |  | **6‘986** |  |  |  |
|  | 2.7 |  | 25.5 |  |  |  |

^+^ only found in LMA treatment; ^‡^ only found in DMA treatment; ^1^ Protein Knowledgebase (UniProtKB); ^2^ Rapala et al. (2005); ^3^ Li et al. (2010); ^4^Van den Wyngaert et al. (2011); * bacterial genus provided genomic reference for MC degradation
